# The Influence of Footwear on the Modular Organization of Running

**DOI:** 10.3389/fphys.2017.00958

**Published:** 2017-11-22

**Authors:** Alessandro Santuz, Antonis Ekizos, Lars Janshen, Vasilios Baltzopoulos, Adamantios Arampatzis

**Affiliations:** ^1^Department of Training and Movement Sciences, Humboldt-Universität zu Berlin, Berlin, Germany; ^2^Berlin School of Movement Science, Humboldt-Universität zu Berlin, Berlin, Germany; ^3^Research Institute for Sport and Exercise Sciences, Liverpool John Moores University, Liverpool, United Kingdom

**Keywords:** muscle synergies, locomotion, running, barefoot running, motor control, EMG, footwear

## Abstract

For most of our history, we predominantly ran barefoot or in minimalist shoes. The advent of modern footwear, however, might have introduced alterations in the motor control of running. The present study investigated shod and barefoot running under the perspective of the modular organization of muscle activation, in order to help addressing the neurophysiological factors underlying human locomotion. On a treadmill, 20 young and healthy inexperienced barefoot runners ran shod and barefoot at preferred speed (2.8 ± 0.4 m/s). Fundamental synergies, containing the time-dependent activation coefficients (motor primitives) and the time-invariant muscle weightings (motor modules), were extracted from 24 ipsilateral electromyographic activities using non-negative matrix factorization. In shod running, the average foot strike pattern was a rearfoot strike, while in barefoot running it was a mid-forefoot strike. In both conditions, five fundamental synergies were enough to describe as many gait cycle phases: weight acceptance, propulsion, arm swing, early swing and late swing. We found the motor primitives to be generally shifted earlier in time during the stance-related phases and later in the swing-related ones in barefoot running. The motor primitive describing the propulsion phase was significantly of shorter duration (peculiarity confirmed by the analysis of the spinal motor output). The arm swing primitive, instead, was significantly wider in the barefoot condition. The motor modules demonstrated analogous organization with some significant differences in the propulsion, arm swing and late swing synergies. Other than to the trivial absence of shoes, the differences might be deputed to the lower ankle gear ratio (and the consequent increased system instability) and to the higher recoil capabilities of the longitudinal foot arch during barefoot compared to shod running.

## Introduction

In the last decade, the study of locomotion in evolutionary anthropology has been increasingly focusing on endurance running. Humans, compared to non-human primates, show exceptional endurance running speeds (Bramble and Lieberman, 2004). However, the advent of modern running shoes is contemporary history compared to the two million-years-old fossil evidence of running as a derived capability of the genus *Homo* (Bramble and Lieberman, 2004; Lieberman et al., 2010). Running barefoot or in minimal footwear has been the predominant condition for most of the human history (Lieberman et al., 2010). Hence, it can be expected that the strategies adopted to run barefoot might differ from those employed to run shod.

During running, the foot can strike the ground in multiple ways, called foot strike patterns (FSPs). Rearfoot (RS), midfoot (MS), and forefoot (FS) strike are the common classifications, depending on the location of the first contact area with the ground (Hasegawa et al., 2007). We recently found that if almost 90% of the population adopts a RS pattern when running shod, only half maintain it when switching to barefoot (Santuz et al., 2016), changing to either MS or FS (joined in an unique pattern and indicated as mid-forefoot strike, MFS for brevity). Adopting MFS patterns can increase the plantarflexors activity, reduce the ground contact times and affect the kinetics and kinematics of the whole gait cycle (Komi, 1984, 1992; von Tscharner et al., 2003; Hasegawa et al., 2007; Lieberman et al., 2010). Therefore, we can argue that switching between the two conditions of running shod and barefoot does not only imply kinematic and kinetic changes, but might involve a different organization of movement. From a motor control perspective, this assumption can be investigated by analyzing the modular organization of muscle activity before and after altering the running condition.

Since the late 1960s (Bernstein, 1967) it has been accepted that the central nervous system can simplify the production of movements by avoiding the activation of each muscle separately (Mussa-Ivaldi et al., 1994; Bizzi et al., 2008). This important feature might be implemented by reducing the degrees of freedom through a linear combination (Mussa-Ivaldi et al., 1994) of specific muscle activation patterns, called synergies (Bizzi et al., 2008). A number of studies were able to show that synergies reside in the brain stem or spinal cord and follow a modular organization (Tresch et al., 2002; Hart and Giszter, 2004; Bizzi et al., 2008; Roh et al., 2011; Bizzi and Cheung, 2013). Recently, a study in mice using optogenetics to isolate the excitatory and inhibitory neuronal populations could show a strong specificity in the spinal cord topography (Hägglund et al., 2013). The synergies as low dimensional units, via descending or afferent pathways, produce a complex electromyographic (EMG) pattern in muscles (Tresch et al., 2002; Bizzi and Cheung, 2013), creating a locomotor drive mediated by a certain amount of supraspinal control (Roh et al., 2011). During walking, the same amount of basic activation patterns could be found in patients with spinal cord injury and in healthy participants at different speeds and gravitational loads (Ivanenko et al., 2006). Synergies similar to those found in humans at a spinal (Ivanenko et al., 2006) or muscular level can be observed also in the motor cortex of the primate and cat (Yakovenko et al., 2011; Overduin et al., 2015). Moreover, studies on the excitability of the corticospinal system showed that training can improve task-specific brain organizations (Moscatelli et al., 2016a,b; Monda et al., 2017). This suggests a high degree of cooperation within the central nervous system's structure at all levels. In this study, we used an unsupervised learning method called non-negative matrix factorization (NMF) (Lee and Seung, 1999) for reducing the high dimensional EMG input into a small number of synergies. We focus on the comparison between the modular organization of shod and barefoot running. Compared to the analysis of direct EMG signals, the muscle synergies concept has the clear advantage of being a high-throughput approach for analyzing muscle activities. In fact, it does not only provide the researcher with an automatic, low-dimensional, clustering of the activations during the gait cycle, but it also identifies the weighted contribution of each muscle for producing a certain movement.

The objective of the current study was to investigate the modular organization of shod and barefoot running using muscle synergies in order to gain new knowledge about the neurophysiological factors underlying human locomotion. Based on reported changes in the kinematic, kinetic (Lieberman et al., 2010) and EMG (Komi, 1984, 1992; von Tscharner et al., 2003; Cappellini et al., 2006, 2010; Ivanenko et al., 2008) characteristics of shod and barefoot running, we hypothesized that there is an alteration in the modular organization between the two conditions that might be associated with the specificity of the respective foot strike with the ground. In a first step we calculated the similarities between trials of the same condition using the coefficient of determination (*R*^2^) and set their repeatability (intraday) thresholds (Santuz et al., 2017). Afterwards, we investigated the similarities between the two conditions and compared with the intraday thresholds. This two-step process allowed an improved objective and quantitative interpretation of the conventionally qualitative output given by methods like the NMF.

## Materials and methods

### Experimental protocol

Twenty healthy and young adults were recruited (10 male, height 180 ± 5 cm, body mass 77 ± 8 kg, age 31 ± 7 years, 10 female, height 169 ± 8 cm, body mass 60 ± 8 kg, age 28 ± 5 years). All the participants were regularly active and did not use orthotic insoles and did not have any previous experience of barefoot running. None showed or reported any history of neuromuscular or musculoskeletal impairments, or any head or spine injury at the time of the measurements or in the previous 6 months. This study was reviewed and approved by the Ethics Committee of the Humboldt-Universität zu Berlin. All the participants gave written informed consent for the experimental procedure, in accordance with the Declaration of Helsinki.

The muscle activity of 24 ipsilateral muscles was recorded using one 16-channel (myon m320, myon AG, Schwarzenberg, Switzerland) and one 8-channel (myon RFTD E08, myon AG, Schwarzenberg, Switzerland) wireless surface-EMG systems. The acquisition frequency was set to 1,000 Hz (16 ms latency, constant). Vertical ground reaction forces (VGRFs) were recorded at 120 Hz through a pressure plate (FDM-THM-S, zebris Medical GmbH, Isny im Allgäu, Germany) integrated with a treadmill (mercury, H-p-cosmos Sports and Medical GmbH, Nussdorf, Germany). The pressure plate data were acquired using the proprietary software (WinFDM-T v2.5.1, zebris Medical GmbH, Isny im Allgäu, Germany) and then extracted in a raw format for autonomous post-processing using a validated custom algorithm (Santuz et al., 2016) written in R version 3.4.1 (R Foundation for Statistical Computing, R Core Team, Vienna, Austria). The EMG devices and the plate were synchronized using an analog signal.

The participants completed a self-selected warm-up on the treadmill, in order to choose their comfortable shod-running speed. The procedure to find the comfortable speed was implemented using the method of limits (Treutwein, 1995). The speed was randomly increased with steps of 0.02 to 0.05 m/s at varying time intervals (around 5 to 10 s) until the participant was comfortable with a specific pace. The operation was then repeated starting from a faster speed and randomly decreasing it as previously done. If the comfortable value did not differ more than 10% from the previous, the average of the two values was taken as the preferred. Otherwise, the whole procedure was iterated. The warm-up protocol typically lasted between 5 and 10 min. After being instructed about the protocol, the participants completed two different tasks, in random order: shod running at the preferred running speed (2.9 ± 0.4 m/s for male, 2.6 ± 0.2 m/s for female) and barefoot running at the same speed.

For each condition, the muscle activity of the 24 ipsilateral (right side) muscles was recorded: *splenius capitis* (SP), *trapezius* (descending, TR), *latissimus dorsi* (LD), *deltoid* (anterior, DA), *deltoid* (posterior, DP), *biceps brachii* (BB), *triceps brachii* (long head, TB), *erector spinæ* (*longissimus*, L1 vertebra, ES), *rectus abdominis* (RA), *abdominal external oblique* (AE), *gluteus medius* (ME), *gluteus maximus* (MA), *adductor longus* (AL), *tensor fasciæ latæ* (FL), *rectus femoris* (RF), *vastus medialis* (VM), *vastus lateralis* (VL), *semitendinosus* (ST), *biceps femoris* (long head, BF), *tibialis anterior* (TA), *peroneus longus* (PL), *gastrocnemius medialis* (GM), *gastrocnemius lateralis* (GL) and *soleus* (SO). Around 50 gait cycles (49 ± 4) (Oliveira et al., 2014) were recorded after an accommodation period of maximum 60 s (White et al., 2002). Between the trials there was a break necessary to change shoes before and after running barefoot. The same randomized protocol was repeated after 15 min of rest for use in the (intraday) repeatability analysis, without removing the electrodes.

### Foot strike patterns assessment

For every trial, the FSP and the strike index were calculated using a validated algorithm based on the numerical analysis of foot pressure distribution (Santuz et al., 2016). As we previously suggested (Santuz et al., 2016), the FSPs have been grouped into two categories rather than three: RS and MFS (including MS and FS patterns). The strike index, as originally defined by Cavanagh and Lafortune (Cavanagh and Lafortune, 1980), was calculated as the distance from the heel to the center of pressure at impact relative to total foot length.

### Spinal motor output assessment

For the spinal motor output characterization, we mapped the 24 measured EMG activities onto the estimated rostrocaudal location of alpha-motoneurons (MNs) pools in the segments from the second cervical vertebra (C2) to the second sacral vertebra (S2) of the spinal cord (Ivanenko et al., 2006; La Scaleia et al., 2014). The segments T2, T3, and T4 have been excluded from the analysis since they do not innervate any of the considered muscles. The wireless EMG systems had a built-in band-pass filter (5–500 Hz, 3 dB/oct, 4th order). The EMG signals were high-pass filtered and then full-wave rectified and low-pass filtered using a 4th order IIR Butterworth zero-phase filter with cut-off frequencies 50 Hz (high-pass) and 20 Hz (low-pass for the linear envelope) using R v3.4.1 (R Found. for Stat. Comp.). The amplitude was normalized to the maximum activation recorded for each participant across all conditions (Karamanidis et al., 2004; Bizzi et al., 2008; Devarajan and Cheung, 2014). Each gait cycle was then time-normalized to 200 points (Cappellini et al., 2016), assigning 100 points to the stance and 100 points to the swing phase. The cervical segments (C2 to C8) mainly innervate upper limb and neck muscles. The thoracic segments (T1 to T12) connect to the trunk muscles, while the lumbar (L1 to L5) and sacral (S1 and S2) segments innervate the lower limb muscles. The contribution of each muscle to the total estimated activity of the spinal segments was implemented using the myotomal charts developed by Kendall et al. (2005). This method shows the organization of the efferent MNs network directed to the muscles, assuming a common spinal topography among the investigated participants. Without accounting for size differences in MN pools at each spinal level, the motor output of each spinal segment *S*_*j*_ was estimated using the Equation (1.1) (La Scaleia et al., 2014):

(1.1) $$S_{j}=\frac{\sum_{i=1}^{m_{j}}\left(\frac{k_{ji}}{n_{i}}\times EMG_{i}\right)}{\sum_{i=1}^{m_{j}}\left(\frac{k_{ji}}{n_{i}}\right)}$$

where *m*_*j*_ are the muscles innervated by each segment, *n*_*i*_ is the number of spinal levels that innervate the *i*th muscle, *k*_*ij*_ is a weighting coefficient specific to each muscle and spinal segment (e.g., *k*_*ij*_ = 1 or *k*_*ij*_ = 0.5 if *S*_*j*_ is a major or minor MN source, respectively) and *EMG*_*i*_ is the normalized recorded EMG, specific for each participant and trial (Kendall et al., 2005; La Scaleia et al., 2014).

### Modular organization assessment

The gait cycle breakdown was obtained from the pressure plate's raw data. Using a custom algorithm (Santuz et al., 2016), the touchdown was identified as the first non-zero pressure matrix after the last toe-off. The EMG signals were pre-processed using the filtering and normalization conditions reported above.

Muscle synergies data were extracted through a custom script (Santuz et al., 2017) (R v3.4.1, R Found. for Stat. Comp.) using the classical Gaussian NMF algorithm (Lee and Seung, 1999) from the first circa 50 gait cycles of each acquisition (Oliveira et al., 2014). EMG data were pre-processed using the same filtering conditions reported in the previous paragraph. The *m* = 24 time-dependent muscle activity vectors were grouped in an *m* × *n* matrix *V*, factorized such that *V* ≈ *V*_*R*_ = *WH*. *V*_*R*_ represents the new reconstructed matrix, which approximates the original matrix. The motor primitives matrix *H* (Dominici et al., 2011; Santuz et al., 2017) contained the time-dependent coefficients of the factorization with dimensions *r* × *n*, where *r* represents the number of synergies necessary to reconstruct the signal and *n* the number of data points (*n* = 200 number of cycles). The motor modules matrix *W* (Gizzi et al., 2011; Santuz et al., 2017) with dimensions *m* × *r*, contained the time-invariant muscle weightings. *H* and *W* described the synergies necessary to accomplish a movement. The update rules for *H* and *W* are presented in Equations (2.1, 2.2). The limit of convergence was reached when a change in the calculated *R*^2^ between *V* and *V*_*R*_ was smaller than the 0.01% in the last 20 iterations (Cheung et al., 2005; Santuz et al., 2017). To choose the minimum number of synergies required to represent the original signals, the curve of *R*^2^ values vs. synergies was fitted using a simple linear regression model, using all 10 synergies. The mean squared error was then repeatedly calculated, each time removing the lower synergy point, until only two points were left or until the mean squared error fell below 10^−5^ (Santuz et al., 2017).

(2.1, 2.2) $$\left\{ \begin{array}{l} H=H\frac{\left(W^{T}V\right)}{\left(W^{T}WH\right)}\\ W=W\frac{\left(VH^{T}\right)}{\left(WHW^{T}\right)} \end{array}\right.$$

**Figure 1:**
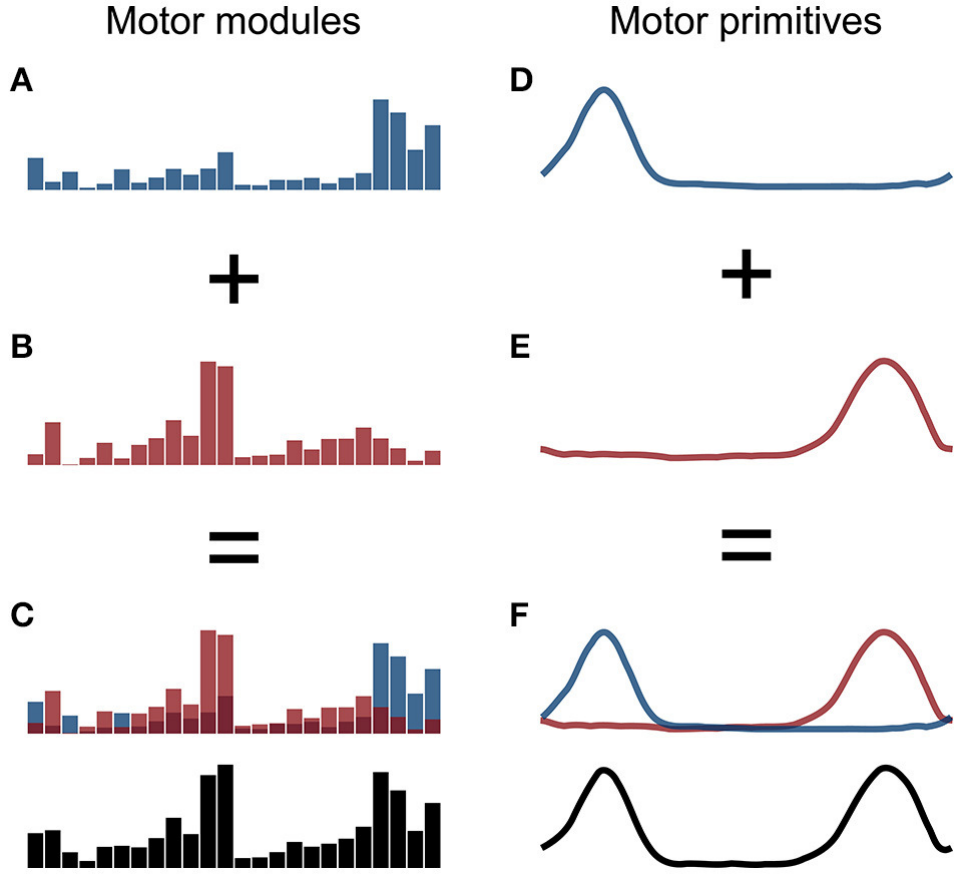
Example of two fundamental synergies combined into one. The histograms in the panels **A,B** represent the two fundamental sets of motor modules and the curves in the panels **D,E** the two respective primitives, with arbitrary x- and y-axis units. The combined motor modules and primitives are shown in panels **C,F**, respectively.

The aforementioned procedure allowed us to extract fundamental and combined synergies from the raw EMG data. A fundamental synergy can be defined as an activation pattern whose motor primitive shows a single peak of activation (Santuz et al., 2017). When two or more fundamental synergies are blended into one, a combined synergy appears. Due to the lack of consent in the literature on how to interpret combined synergies, they were excluded from the analysis. An example of combined synergies is reported in Figure 1.

The fundamental synergies recognition was implemented using a custom learning algorithm based on a curve-fitting model (Santuz et al., 2017). The first implementation step consists in choosing some examples of single-peaked activation patterns, which might represent a fundamental primitive. The code is then provided with this training set and a search of similar shapes is done across the whole dataset of factorized curves. With a first iteration, the primitives that have a high similarity (*R*^2^ > 0.95) with the ones present in the training set are added to it. The number of fundamental primitives is then selected by clustering similar motor modules. After updating the training set, the code starts the recognition across the entire dataset searching, synergy-by-synergy, for similar primitives (we found *R*^2^ > 0.50 to be a good threshold). Non-recognized curves can then be visually inspected with an interactive routine or automatically identified as new fundamental or combined primitives. This approach, validated in a pilot study, can reproduce the results of a completely manual selection of the curves with a margin of error of ± 5%.

### Metrics for comparison of curves

We evaluated the center of activation (*CoA*) and full width half maximum (FWHM) for the resulting curves of the extracted spinal maps and motor primitives (matrix H) in both conditions and types of locomotion. The *CoA* was defined as the angle of the vector (in polar coordinates) that points to the center of mass of that circular distribution (Cappellini et al., 2016). The polar direction represented the gait cycle's phase, with angle 0 ≤ θ_*t*_ ≤ 2π. The following equations define the *CoA*:

(3.1, 3.2) $$\left\{ \begin{array}{l} A=\sum_{t=1}^{p}\left(\cos\theta_{t}\times P_{t}\right)\\ B=\sum_{t=1}^{p}\left(\sin\theta_{t}\times P_{t}\right) \end{array}\right.$$

(3.3) CoA=arctan(B/A)

where *p* is the number of points of each gait cycle (*p* = 200) and *P* is the activation vector. The FWHM was calculated as the number of points exceeding each gait cycle's half maximum, after subtracting the gait cycle's minimum (Cappellini et al., 2016). For every trial, both parameters were calculated at each gait cycle and then averaged to proceed with the statistical analysis. A maximum of 50 gait cycles for each acquisition were selected for analysis. The *CoA* and FWHM were analyzed for stance and swing distinctively for spinal maps and over the whole gait cycle for the motor primitives.

### Statistics

A two-way ANOVA with repeated measures, followed by a Tukey *post-hoc* analysis with false discovery rate *p-*value adjustment, was used to investigate *CoA* and FWHM between conditions. The same statistics was used for the motor modules, using the muscles and the conditions (shod vs. barefoot) as independent variables. To assess the similarities between the fundamental motor primitives of shod and barefoot running, we used the coefficient of determination *R*^2^. We calculated the similarity values between the pairs of trials recorded during the same day (intraday repeatability) in shod and barefoot running. Then, we compared them with the similarity values between the two conditions (shod and barefoot running). Type A uncertainty was expressed as *u*_*A*_ = *s*/_√_*n*. All the significance levels were set to α = 0.050 and the statistical analyses were conducted using R v3.4.1 (R Found. for Stat. Comp.).

## Results

### Foot strike patterns and gait parameters

**Figure 2:**
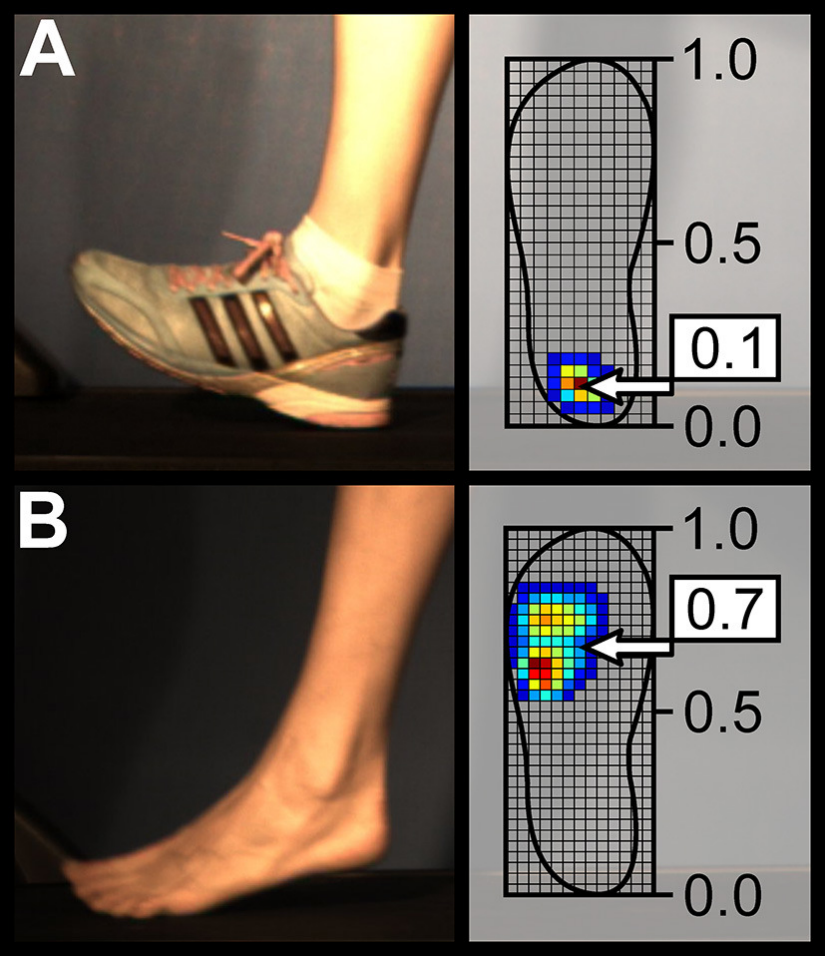
Sagittal view of a typical rearfoot **(A)** and forefoot **(B)** strike patterns during shod and barefoot running, respectively. The strike index values extracted from plantar pressure distribution for these two representative cases are presented as well.

Out of 20 participants, 14 (7 male, 7 female) transitioned from RS (shod) to MFS (barefoot). Three kept the MFS pattern in both conditions and three retained a RS pattern in both shod and barefoot running. The participants demonstrated significant (*p* < 0.001) differences in the average strike index, presenting values of 0.15 ± 0.17 in shod running and 0.53 ± 0.18 in barefoot running (with 0 denoting the most posterior and 1 the most anterior point of the shoe, see Figure 2). Also the average contact times of 301 ± 36 ms and 274 ± 32 ms as well as the average cadence (step frequency) of 162 ± 10 and 166 ± 11 steps/min were significantly different (*p* < 0.001) between shod and barefoot running, respectively. The mean values of the left and right VGRFs normalized to body weight were significantly lower in the barefoot condition (1.82 ± 0.20 for the shod and 1.75 ± 0.16 for the barefoot condition, *p* < 0.001). The impulse (mean values of left and right sides) was significantly lower in the barefoot condition (201 ± 39 N·s vs. 186 ± 35 N·s, *p* < 0.001), but the flight time was contrarily higher (70 ± 24 ms for shod and 89 ± 22 ms for barefoot running, *p* < 0.001).

### Spinal motor output

**Figure 3:**
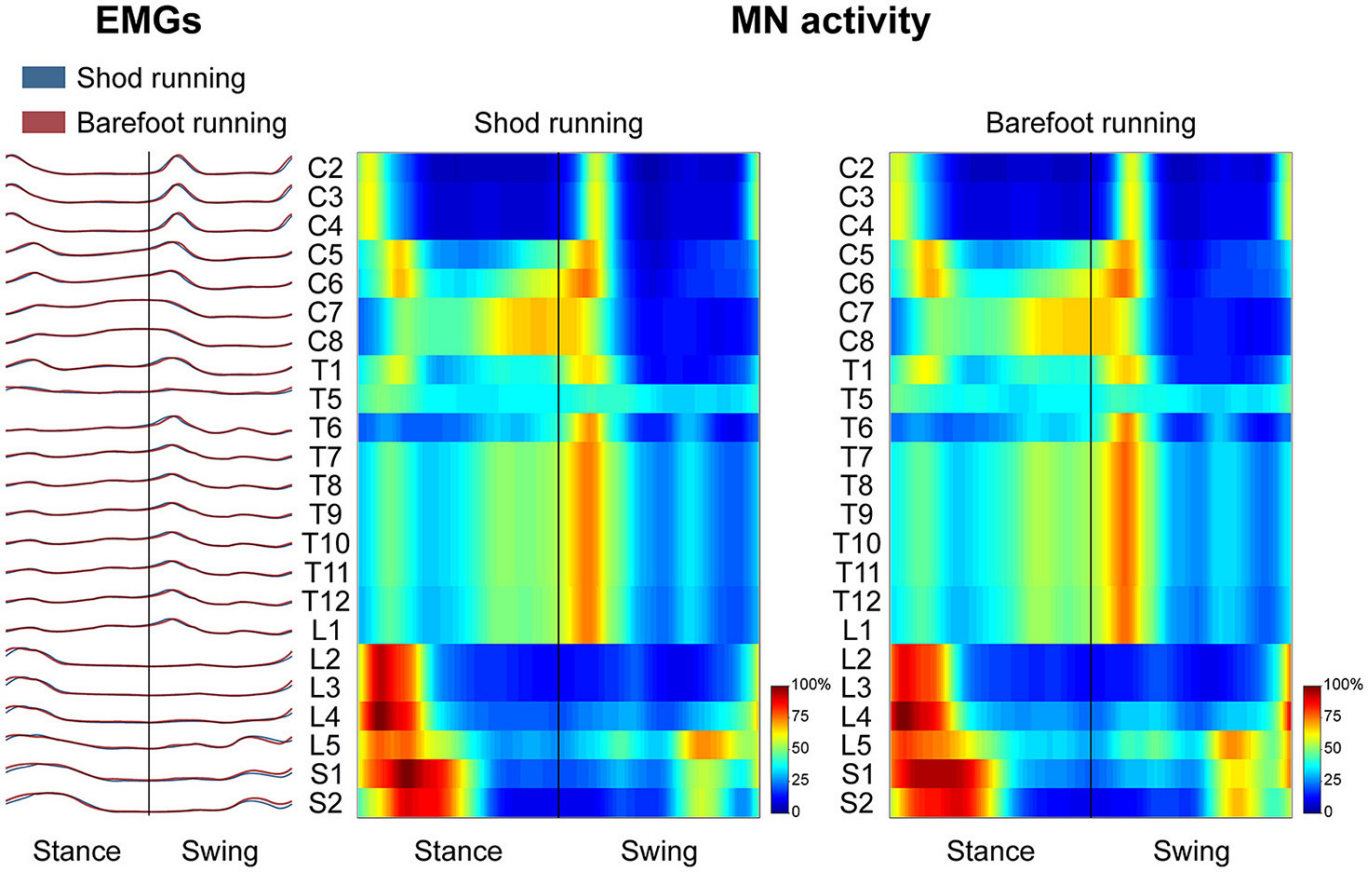
The average spatiotemporal spinal motor output is presented for shod and barefoot running, normalized in amplitude to the maximum of each segment. These curves have been obtained by mapping each of the 24 muscle activations onto the relative spinal segment (cervical from C2 to C8, thoracic from T1 to T12, lumbar from L1 to L5 and sacral from S1 and S2). The two level plots show the average alpha-motoneurons activity for each condition, giving additional information about the absolute activation level (normalization to the maximum of each condition). The stance and swing phases have been temporally normalized to the same amount of data points (100 each). Values are the means across all subjects and all trials.

Figure 3 depicts the average spatiotemporal spinal motor output for shod and barefoot running. The two-way ANOVA identified statistically significant differences in the FWHM of the mapped EMG activities when comparing shod and barefoot running for both the stance (*p* = 0.018) and swing (*p* = 0.019) phase of the gait cycle (Table 1). The *post-hoc* analysis showed significantly lower FWHM in the barefoot condition of segment L4's spinal motor output, innervating the muscles ME, AL, FL, RF, VM, VL, ST, TA, and PL. The *CoA* was not significantly different between conditions in neither the stance (*p* = 0.107) or the swing (*p* = 0.091) phase (Table 1).

**Table 1:** Differences between shod and barefoot running in the center of activity (*CoA*) and full width at half maximum (FWHM) of the electromyographic activities mapped onto the estimated rostrocaudal location of the spinal cord (segments C2 to S2).

**Segment**	***CoA***		**FWHM**			
	**Stance *p* = 0.107**	**Swing *p* = 0.091**	Stance *p* = 0.018^*^		Swing *p* = 0.019^*^	
	**Δ_S,B_(%)**	**Δ_S,B_(%)**	**Δ_S,B_(%)**	***p-*value**	**Δ_S,B_(%)**	***p-*value**
C2	+2.6	+2.7	−0.8	0.718	−0.8	0.712
C3	+2.5	+2.5	−1.0	0.658	−1.0	0.654
C4	+2.5	+2.5	−1.0	0.658	−1.0	0.654
C5	−0.2	−0.2	−0.9	0.698	−0.9	0.669
C6	−0.5	−0.5	−0.6	0.794	−0.6	0.818
C7	−0.4	−0.4	−1.7	0.362	−1.6	0.403
C8	−0.4	−0.4	−1.7	0.362	−1.6	0.403
T1	+0.4	+0.5	−1.0	0.657	−1.1	0.614
T5	+3.0	+2.9	−1.7	0.371	−1.8	0.342
T6	−0.9	−0.9	+0.2	0.949	+0.2	0.959
T7	+0.0	+0.0	+0.2	0.954	+0.2	0.930
T8	+0.0	+0.0	+0.2	0.954	+0.2	0.930
T9	+0.0	+0.0	+0.2	0.954	+0.2	0.930
T10	+0.0	+0.0	+0.2	0.954	+0.2	0.930
T11	+0.0	+0.0	+0.2	0.954	+0.2	0.930
T12	−0.2	−0.2	0.1	0.984	0.1	0.972
L1	−0.2	−0.2	0.1	0.984	0.1	0.972
L2	+1.7	+1.9	−2.3	0.210	−2.3	0.212
L3	+1.7	+1.9	−2.3	0.210	−2.3	0.212
L4	+0.2	+0.2	−*3.7*	0.036^*^	−*4.0*	0.025^*^
L5	−0.6	−0.6	−2.1	0.252	−2.3	0.209
S1	+0.5	+0.6	−0.1	0.995	+0.0	0.996
S2	+0.6	+0.6	1.0	0.641	1.0	0.630

*Results concerning T2, T3 and T4 are not reported since those segments do not innervate any muscle considered in this study. Positive differences (Δ_E,U_ >0) denote bigger values in the barefoot condition, whereas negative differences imply lower values*.

### Modular organization

**Figure 4:**
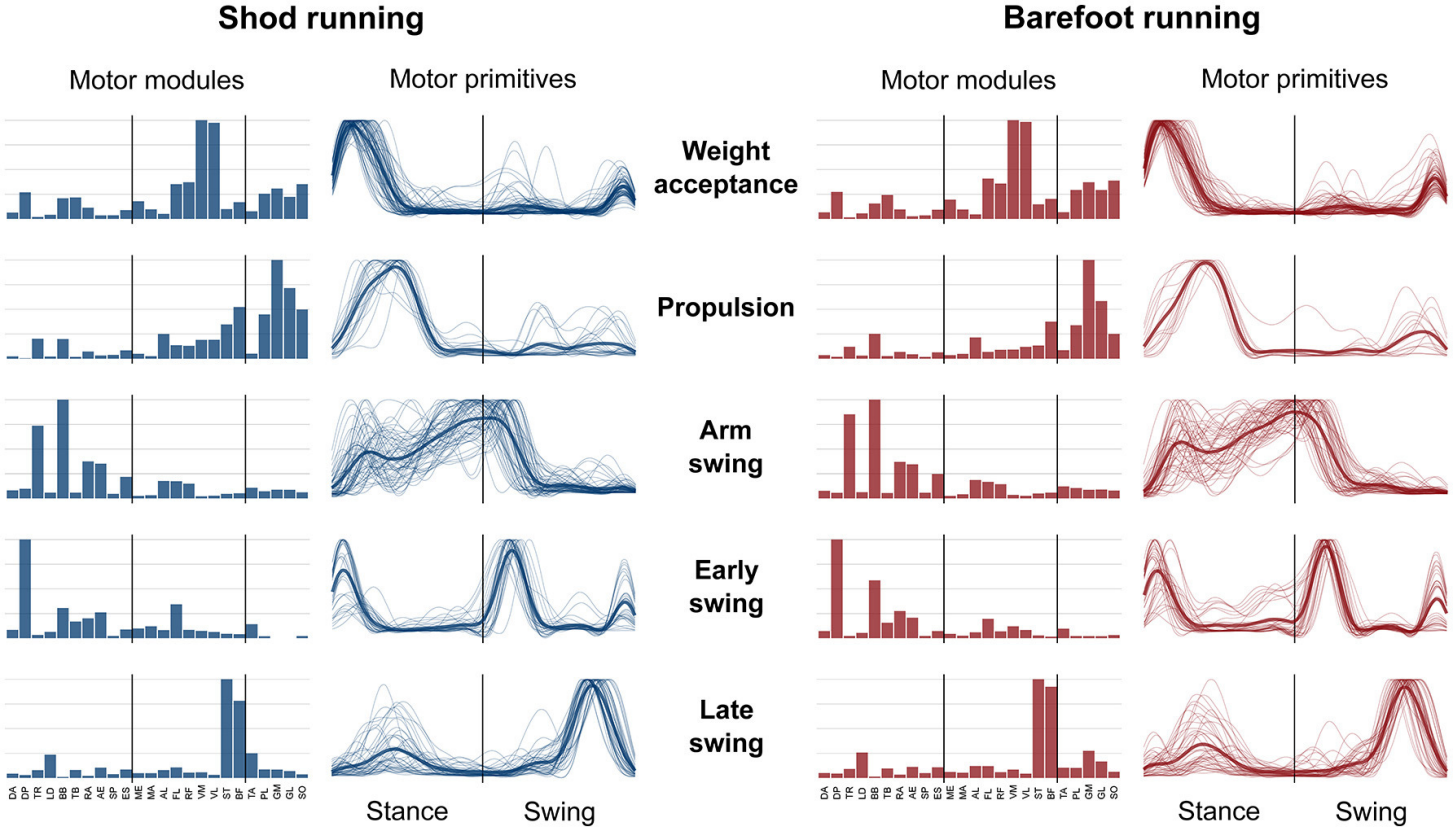
Motor modules and motor primitives for shod and barefoot running at the comfort speed. The motor modules are presented on a normalized y-axis base. For the motor primitives, the x-axis full scale represents one gait cycle (stance and swing normalized to the same amount of points and divided by a vertical line) and the y-axis the normalized amplitude. The motor modules represent the average normalized values across all the participants. The mean motor primitives are represented with a thick black line, while all the trials are denoted by thin gray lines.

**Figure 5:**
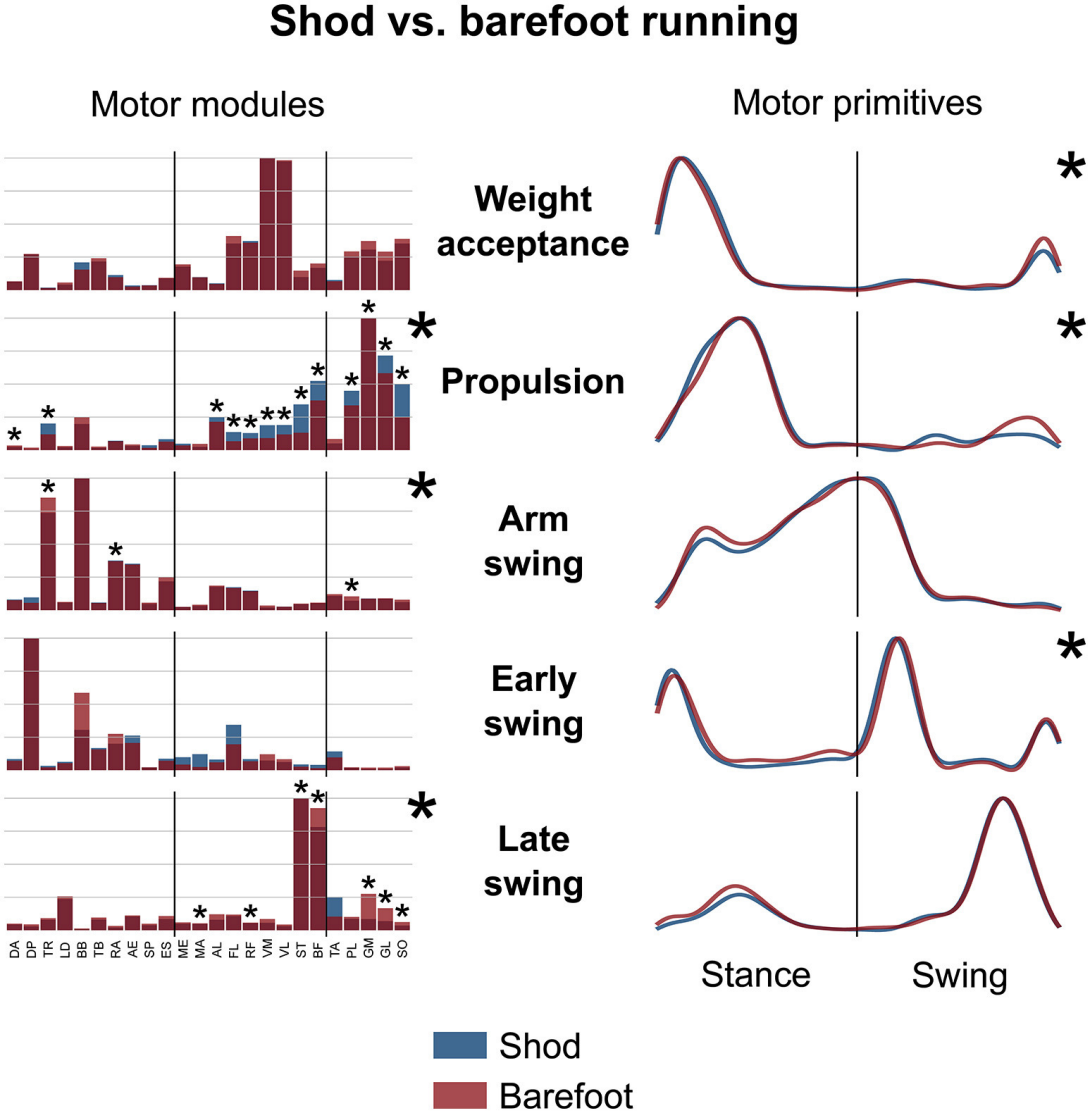
Average motor modules and motor primitives of the five fundamental synergies for shod and barefoot running at the comfort speed. The motor modules are presented on a normalized y-axis base. For the motor primitives, the x-axis full scale represents one gait cycle (stance and swing normalized to the same amount of points and divided by a vertical line) and the y-axis the normalized amplitude. Asterisks denote significant differences between shod and barefoot running.

The average number of recognized fundamental synergies during running was significantly different between the two conditions (3.9 ± 0.6 for shod and 3.6 ± 0.6 for barefoot running, *p* < 0.001). However, in both conditions, five fundamental activation patterns could be identified (Figures 4, 5). The five fundamental synergies extracted during both shod and barefoot running, were associated with temporally different phases of the gait cycle and ordered according to the timing of each motor primitive's global maximum (Figures 4, 5). The first synergy (peak at ~8% of the stance phase) functionally referred to the body weight acceptance, with a major involvement of knee extensors and plantarflexors. The second synergy (peak at ~27% of the stance phase) described the propulsion phase, to which the plantarflexors mainly contributed. The third synergy (peak at ~90% of the stance phase) was associated with the arm swing, when the upper body muscles played an important role. The fourth synergy (peak at ~22% of the swing phase) identified the early swing, showing contributions from upper body muscles, stabilizing muscles of the lower limb and the start of foot dorsiflexors activation. The fifth and last synergy (peak at ~72% of the swing phase) reflected the late swing and the landing preparation, highlighting the relevant contribution of knee flexors, foot dorsiflexors (in the shod condition) and plantarflexors (in the barefoot condition).

The motor primitives of the weight acceptance, propulsion and early swing synergies were significantly dissimilar (*p* = 0.023, 0.002, and <0.001, respectively; Figure 5, Table 2). The motor modules exhibited significant differences in the propulsion (*p* < 0.001), arm swing (*p* = 0.023) and late swing (*p* < 0.001) synergies (Figure 5). The muscles responsible for said changes where mainly the upper and lower leg muscles in the propulsion (higher contribution in the shod condition), the trunk muscles in the arm swing (higher contribution in the barefoot condition), the knee flexors and foot plantarflexors in the late swing synergy (higher contribution in the barefoot condition, Figure 5).

**Table 2:** Similarities, indicated as $R_{\text{S},\text{B}}^{2}$, between the motor primitives of shod and barefoot running as mean of intraday repetitions.

	**Motor primitives**		
	***R*^2^_S,B_**	***R*^2^_S,B_ intraday**	***p-*value**
Weight acceptance	*0.87 ± 0.15*	*0.92 ± 0.11*	0.023^*^
Propulsion	*0.91 ± 0.08*	*0.92 ± 0.21*	0.002^*^
Arm swing	0.77 ± 0.35	0.82 ± 0.24	0.785
Early swing	*0.82 ± 0.24*	*0.89 ± 0.25*	<0.001^*^
Late swing	0.90 ± 0.09	0.88 ± 0.15	0.837

*The intraday repeatability values are reported as mean of four trials (two shod and two barefoot). Values ± Type A uncertainty. The p-values were calculated by comparing the R^2^ between shod and barefoot running and the R^2^ for intraday trials*.

The *CoA* of the motor primitives for all the synergies, except from the early swing one, moved significantly in time. The *CoA* values were lower in barefoot running (anticipated activation) for those synergies related completely or partially to the stance phase. For those synergies describing the only swing phase, the *CoA* values were instead bigger in the barefoot compared to the shod condition (Table 3). Further, we found a significant (*p* < 0.001) decrease in the FWHM of the propulsion primitives and an increase (*p* < 0.001) of the arm swing primitives in barefoot compared to shod running (Table 3).

**Table 3:** Differences between shod and barefoot running in the center of activity (*CoA*) as well as in the relative full width at half maximum (FWHM) of motor primitives.

	**Motor primitives**			
	***CoA***		**FWHM**	
	**Δ_S,B_(%)**	***p-*****value**	**Δ_S,B_(%)**	***p-*****value**
Weight acceptance	−*1.3*	<0.001^*^	+3.2	0.174
Propulsion	−*1.3*	<0.001^*^	−*6.2*	<0.001^*^
Arm swing	−*0.9*	0.014^*^	+*20.2*	<0.001^*^
Early swing	+0.5	0.271	+1.9	0.135
Late swing	+*1.2*	0.008^*^	+4.6	0.554

*Positive differences (Δ_S,B_ >0) denote bigger values in the barefoot condition, whereas negative differences imply lower values*.

## Discussion

In this study, we analyzed the modularity of the neuromuscular control of shod and barefoot running. We hypothesized a different modular organization of motion mainly due to the presence or absence of shoes in the two conditions. We found that the motor primitives (or fundamental activation patterns) were generally shifted earlier in time during the stance-related phases and later in the swing-related ones. The motor primitives were found to be significantly wider in the arm swing phase but not in the propulsion, where the basic activation was significantly of shorter duration (peculiarity confirmed by the analysis of the spinal motor output). Moreover, the motor modules (or muscle weightings) demonstrated analogous organization with some significant differences in the propulsion, arm swing and late swing synergies.

The cadence and the strike index significantly increased when changing from shod to barefoot running. Contact times and VGRFs decreased accordingly in the barefoot compared to the shod condition. These results agree with previous studies (De Wit et al., 2000; Lieberman et al., 2010) on the comparison of shod and barefoot running. It is well known that the gear ratios of the ankle joint muscles [i.e., the ratio between the ground reaction force and the muscle force moment arms Carrier et al., 1994] do not only vary through the running stance phase (Carrier et al., 1994), but also when switching from the shod to the barefoot condition (Braunstein et al., 2010). In the last 20% of the stance phase the gear ratio at the ankle joint is lower during barefoot compared to shod running (Braunstein et al., 2010). Lower gear ratios at the ankle joint decrease the contact time while running (Lee and Piazza, 2009) and provide an explanation for the shorter contact times found during barefoot running. Further, a lower gear ratio at the ankle joint induces a reduction in the potential of the plantarflexors to generate efficient muscle force due to the force-velocity relationship (Carrier et al., 1994). In inexperienced runners, this may initiate a dynamic instability in the whole system (including the upper body), requiring stabilization achieved through feedback- as well as predictive-based motor control. We recently found a significant decrease in the dynamic stability of running by switching from shod to barefoot (Ekizos et al., 2017). Moreover, it has been reported that the intrinsic foot muscles show higher absolute activation levels during stance in shod compared to barefoot running (Kelly et al., 2016). This difference produces an alteration in the longitudinal arch compression during the stance phase, leading to higher recoil capabilities in barefoot running (Kelly et al., 2016). This increase in the capacity of the foot to store and return energy is likely an odd feature for the unexperienced barefoot runner and might be another mechanism driving the system to an increased instability.

These very same factors (i.e. different gear ratios, dynamic stability and foot's recoil capabilities) could as well partly explain the differences we found in the duration of the motor primitives. First of all, the reduction in duration of the propulsion-related primitive might be a direct consequence of the lower gear ratios and, possibly, of the increased energy storage and return capabilities when running barefoot. However, this does not explain the increase in the duration of the motor primitives in the arm swing synergy. It has been recently shown that the FWHM of EMG activity undergoes, during gait, a systematic decrease with age in typically developing children (Cappellini et al., 2016). Conversely, very limited age-related changes appear in children affected by cerebral palsy. Moreover, cerebral palsy and typically developing children show a comparable structure of motor modules (Cappellini et al., 2016). Analogously, a widening of the motor primitives can be found in adult patients with cerebellar ataxia and in healthy adults walking on a narrow beam and on slippery ground (Martino et al., 2015). This consolidation of the motor output, promoted by learning and impaired by pathology, might reflect the system's need of adding fail-safe robustness to cope with previously unexperienced running conditions (e.g., the absence of footwear).

Concerning motor modules, significant differences were found in the propulsion, arms swing and late swing synergies. The modules of the propulsion phase indicated that upper leg muscles and, most importantly, foot plantarflexors mainly contributed to the inequality. The relative contributions of these muscles were lower in the barefoot condition, indicating a higher specificity of the muscles more important for the propulsion. During arm swing, the TR, RA, and PL muscles were found to be significantly responsible for the identified changes. The relative contribution of TR and PL was higher in barefoot compared to shod running, while the contrary emerged for the RA. However, the intrinsic variability of this synergy's patterns is high and the EMG activities low compared to other gait cycle phases. Therefore, small adaptations in the strategy might translate in statistical differences. The changes in FSP are the cause for the alteration of the motor modules of foot dorsiflexors and plantarflexors in the late swing synergy. In agreement with the prediction based on one of our earlier studies (Santuz et al., 2016), 70% of the participants changed FSP without undergoing a specific training intervention when switching from shod to barefoot running. Most of the times participants automatically switched from RS (shod) to MFS (barefoot). In some cases, participants changed FSP after a few steps, reportedly due to the discomfort of striking the ground with the bare rearfoot. Specifically, 14 out of 20 participants transitioned from RS (shod) to MFS (barefoot). It is well known that the muscles TA and GM and GL play an important role in the final part of the swing phase, just before touchdown (Komi, 1984). In RS patterns, the TA has the twofold task of dorsiflexing the foot to prepare it for the strike and to control the plantarflexion immediately after the touchdown (von Tscharner et al., 2003). In MFS patterns, given the substantial impact loads at contact during running, a preactivation happens right before the strike and the subsequent activation in the early stages of the stance phase (Komi, 1992). Looking at the late swing synergy, it is evident that the TA contributed more in shod running, an activity that mostly involves a RS pattern. In contrast, the average pattern in barefoot running was a MFS, where the preactivation of GM and GL is predominant. These considerations might as well be extended to overground running, since it has been recently shown that treadmill and overground running share similar motor modules with minimal temporal shifts in the motor primitives (Oliveira et al., 2016).

We cannot exclude that habitual barefoot runners might be able to compensate for the differences in the modular organization of muscle activation found in our participants (which were all inexperienced barefoot runners). Although some effects of barefoot running habituation on FSP can be expected, we argue that the main alterations in the motor modules would remain visible in habitual barefoot runners. This mainly because of the predictable changes in the EMG activity (Komi, 1984, 1992; von Tscharner et al., 2003) and, consequently, in the motor modules associated to the kinematic and kinetic alterations induced by a MFS compared to a RS. Concerning motor primitives, however, we suggest that a training intervention focused on the practice of barefoot running might lead to an improvement in the accuracy of motor commands' timing, thus reducing the FWHM of those primitives that here appear wider. Given the characteristics of barefoot running that we discussed above, however, we do not expect that a retraining program would be able to affect consistently the propulsion motor primitive.

With this study using the muscle synergies concept, we bring new insights in the modular organization of shod and barefoot running. Investigating the differences between the synergies, we could confirm that although in both shod and barefoot running five fundamental synergies are enough to describe the running task, a dissimilarity exists in the modular organization of movement. Moreover, we found an increase in the FWHM of the motor primitives of the arm swing synergy: a possible indication of weak motor learning (Cappellini et al., 2016). These findings suggest a reorganization of the motor output possibly due to the nervous system's effort to cope with the biomechanical specificity of barefoot running. This specificity might be explained by a lower ankle gear ratio (Carrier et al., 1994), different FSP (Komi, 1984, 1992; von Tscharner et al., 2003) and increased instability (Ekizos et al., 2017) created by the absence of shoes. The results indicate a possible reorganization of movement when task's complexity either increases or is not well mastered. The required adjustments seem to go in the direction of an improved robustness of motor output guaranteed by longer activation patterns applied on similar muscle modules, showing some adaptability in such a task-specific structure as the muscle synergies.

## Author contributions

Conceptualization: AS, LJ, AE, and AA; Methodology: AS, LJ, and AA; Investigation: AS, LJ, and AE; Formal Analysis: AS and LJ; Writing – Original Draft: AS; Writing – Review and Editing: AS, LJ, AE, VB, and AA; Visualization: AS; Supervision: VB and AA.

### Conflict of interest statement

The authors declare that the research was conducted in the absence of any commercial or financial relationships that could be construed as a potential conflict of interest.
